# Effects of frailty and walking speed on gait variability in older adults

**DOI:** 10.3389/fmed.2026.1785926

**Published:** 2026-03-23

**Authors:** Wenping Zhang, Jiqin Tang, Wenjing Zhang, Wenfei Yu, Yang Hu, Wei Wang, Liduan Wang

**Affiliations:** 1 School of Rehabilitation Medicine, Shandong Second Medical University, Weifang, China; 2 School of Rehabilitation Medicine, Shandong University of Traditional Chinese Medicine, Jinan, China; 3 Department of Neurology, Yiyuan County Traditional Chinese Medicine Hospital, Zibo, China; 4 School of Basic Medical Sciences, Shandong Second Medical University, Weifang, China

**Keywords:** continuous relative phase, coordination variability, endpoint variability, frailty, interlimb, intralimb

## Abstract

**Background:**

Gait variability is crucial for predicting frailty. This study aims to explore the effects of frailty and speed on endpoint variability and coordination variability (CV) during walking.

**Methods:**

Forty-seven adults (≥60 years; 18 males, 29 females) participated in this study. Gait kinematics were captured using Xsens MVN inertial sensors at three speeds (preferred, fast, and slow). Endpoint variability was evaluated using standard deviations (SD) of support times (total, single, and double). The coordination of the lower limb during braking phase and propulsive phase was quantified by continuous relative phase (CRP) analysis for interlimb (hip-hip, knee–knee, ankle-ankle) and intralimb (hip-knee, knee-ankle), and then CV was assessed through SD of interlimb CRP and intralimb CRP. Effect sizes were calculated using partial eta squared (η_p_^2^).

**Results:**

The results revealed an interaction between frailty and speed on total support time variability (*p* = 0.044, η_p_^2^ = 0.116), knee-ankle CV during braking phase (*p* = 0.020, η_p_^2^ = 0.123), and hip-hip CV during propulsive phase (*p* = 0.002, η_p_^2^ = 0.170). Compared to the robust group, the frail group (*p* = 0.036) and/or pre-frail group (*p* = 0.011) exhibited greater total support time variability during fast walking, but lower knee-ankle (*p* < 0.018), hip-hip CV during fast walking (both *p* < 0.001). Compared with other speeds, the total support time variability of fast walking (frail: *p* = 0.015; pre-frail: *p* < 0.001) and knee-ankle CV of preferred speed walking (frail: *p* = 0.016; pre-frail: *p* = 0.017) were greatest for frail and pre-frail groups. Frailty significantly decreased CV, with frail and pre-frail groups demonstrating lower hip-hip (*p* < 0.001, η_p_^2^ = 0.933), ankle-ankle CV (*p* = 0.014, η_p_^2^ = 0.175) during braking phase and knee–knee (*p* < 0.001, η_p_^2^ = 0.837), ankle-ankle CV during propulsive phase (*p* < 0.001, η_p_^2^ = 0.511). Walking speed differentially affected CV without showing a consistent pattern. Specifically, the CV of preferred speed was the greatest for hip-knee during propulsive phase (*p* = 0.011) and ankle-ankle during braking phase (*p* < 0.012), while the CV of fast walking was the greatest for knee–knee during propulsive phase (*p* = 0.006).

**Conclusion:**

Frail elders exhibited greater endpoint variability but smaller CV, especially interlimb CV. During preferred speed walking, frail and pre-frail elders may demonstrate more flexible distal joint coordination, while rigid distal joint coordination during braking phase may increase instability risks during fast walking. This study used a cross-sectional design with a limited sample; future longitudinal studies are needed for verification, and more gait parameters and population differences should be explored.

## Introduction

Aging has emerged as a global challenge. According to the World Health Organization, the global population over 60 will nearly double from 12 to 22% between 2015 and 2050 (1). Frailty, a prevalent geriatric syndrome (2), is characterized by declining physiological function and heightened vulnerability to stressors and diseases (3), leading to adverse outcomes such as reduced quality of life, functional impairment, greater fall risk, frequent hospitalizations, and higher mortality rates (4–6). Current assessment tools of frailty include the Fried Frailty Index (FFI) (3), Frailty Index (FI) (7), Kihon Checklist (KCL) (8), and Clinical Frailty Scale (CFS) (9), among others. The KCL, a self-assessment tool validated for Asian populations, evaluates six domains (activities of daily living, physical function, nutrition, oral health, cognition, and mood) and offers high sensitivity, specificity, and practicality for community use (8). Existing research indicated that pre-frailty and frailty can be reversed through appropriate interventions (3, 10, 11). Therefore, the early identification and prediction of frailty are of critical importance.

Gait is a key indicator of physical function and health in older adults, providing a novel approach for identifying and predicting frailty (12). Studies suggested that gait kinematic assessments can serve as an effective clinical tool for screening frailty in older adults (13–17). Compared to robust older adults, frail individuals exhibited distinct gait alterations, including reduced speed, shorter step length, prolonged double support time, and greater hip flexion angle at toe-off (13). These changes directly reflect the effect of frailty on walking patterns in older adults. With advancing age, the prevalence of frailty increases (2). Moreover, the severity of frailty is associated with gait variability (17–20), which is a measure of fluctuations across multiple gait cycles, reflecting walking consistency and stability (21, 22). Notably, evidence suggested that variability may be a better indicator of gait abnormalities than the average values of gait parameters (14, 22, 23) and has gained attention for its role in assessing motor control and predicting health risks (24).

Hamill et al. classified gait variability into endpoint variability and coordination variability (25). Endpoint variability refers to fluctuations in spatiotemporal parameters like step width, gait speed, and stance time and reflects movement control precision. Lower endpoint variability may suggest higher precision in movement control, whereas higher endpoint variability may indicate reduced precision or greater execution errors (26). Previous studies showed that frail adults exhibited greater stride time variability (14, 19), implying that frailty may lead to decreased control precision and greater instability during walking. Coordination variability assesses fluctuations in the relative motion between joints (27–29). From the perspective of dynamical systems theory, coordination variability represents movement adaptability and flexibility to adapt to task and environmental changes (30). Excessively low variability may lead to rigidity, compromising the system’s ability to adjust to complex environments (30–32). A previous study showed that during specific gait phases (e.g., the initial swing phase of the knee-hip joint), healthy older adults exhibited reduced lower limb joint coordination variability compared to younger adults (33). This suggested that older adults had decreased adaptability and flexibility when responding to task and environmental changes (33).

Walking speed, a fundamental indicator of walking ability, has been shown to be closely associated with gait variability (34–38). Previous studies have reported that walking speed influenced both endpoint variability (39) and coordination variability (38, 40), suggesting that speed may be an important factor modulating gait variability patterns.

While gait variability may be an important predictor of frailty (19) and a promising indicator for distinguishing gait impairments between frailty categories (41), the relationships between frailty, endpoint variability, and coordination variability remain unclear. Therefore, the objective of this study was to investigate the effect of frailty status on endpoint variability and coordination variability during walking. The study hypothesized that frailty status differentially affects endpoint variability and coordination variability, with frail older adults exhibiting greater endpoint variability but reduced coordination variability compared to robust older adults. Additionally, the study also hypothesized that walking speed may influence gait variability. Specifically, fast walking increases endpoint variability, while coordination variability varies depending on the type of measure used.

## Methods

This study was approved by the Institutional Review Board of Shandong Second Medical University (2024YX061). This study was a cross-sectional study. All subjects read and signed an approved institutional review board informed consent form before testing.

### Participants

**Figure 1:**
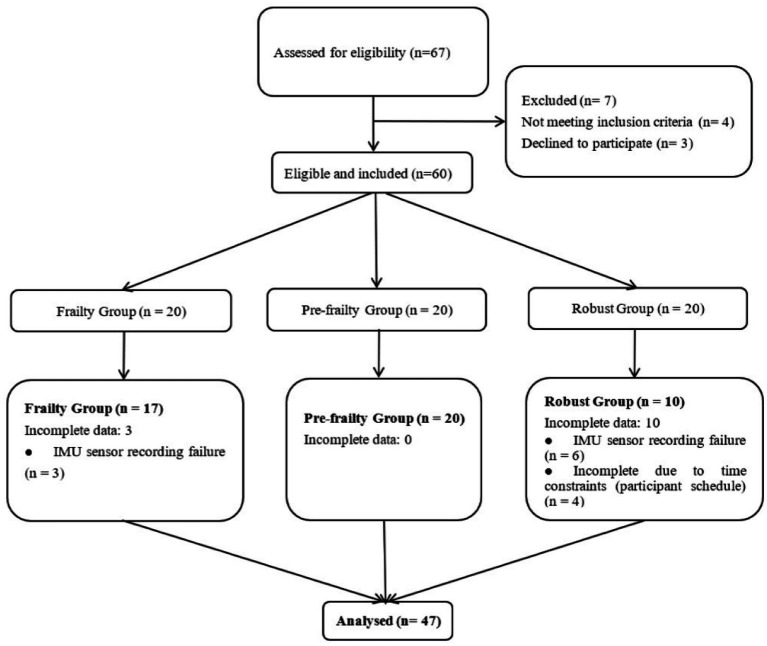
Flowchart for participant recruitment and group allocation.

This was a cross-sectional study using a convenience sample of older adults aged 60 years and older recruited from communities and nursing homes. The inclusion criteria were as follows: (1) the ability to walk independently for more than 10 meters; (2) the absence of significant neuromuscular disorders; and (3) clear consciousness, the ability to complete questionnaires independently, and effective communication with the investigators. The exclusion criteria included: the presence of neurological diseases, severe joint disorders, significant hearing impairment, or advanced-stage cancer. Our final sample consisted of 25 participants from the community and 22 from nursing homes. The detailed screening process was shown in Figure 1.

KCL was used to assess the subjects’ frailty status (8). According to the KCL scoring criteria, a score of 0–3 indicated a robust status, 4–7 indicated a pre-frail status, and ≥8 indicated a frail status (42, 43).

### Data collection

Xsens MVN BIOMECH Awinda (Xsens Technologies BV, Enschede, Netherlands) was used to capture the kinematic characteristics of the lower limbs of the subjects during walking at three different speeds. The Xsens was a motion capture system based on inertial measurement units (IMU) (44). The sampling rate of the IMU system was 100 Hz. Compared to optical motion capture systems, the IMU enabled precise estimation of joint angle time series under various walking conditions and offered high portability, making it suitable for use in natural environments outside the laboratory (45). Multiple authors also confirmed the effectiveness of Xsens in measuring gait spatiotemporal parameters and kinematics (46–48).

Before the experiment, all subjects performed a 10 min warm-up activity. The 10 min warm-up consisted of low-intensity, self-paced walking and joint mobility exercises. All activities were demonstrated by the researcher. Participants were encouraged to perform movements within their comfort level, and the warm-up was considered complete based on their subjective sense of readiness. Rest was permitted at any time. The adaptive protocol was purposefully designed to ensure the safety and comfort of all participants. According to the MVN user manual guidelines (49), seven sensor units were attached over the lower back (lumbar spine, L3–L4), both thighs (lateral middle thigh), both shanks (tuberosities tibiae), and both feet (dorsum of foot). Prior to the formal test, subjects walked for 30 s to familiarize themselves with the test procedure and to complete the calibration of the device. After that, subjects were asked to walk along a 10-meter walkway and complete the walking test at three randomly assigned speeds: preferred, fast, and slow. The instructions for each speed were as follows: (1) walk at your normal daily walking pace, (2) walk as if you are in a hurry to reach the bathroom while ensuring safety, and (3) walk at a leisurely pace as if you are taking a stroll. These context-based instructions were adapted from previous studies (50, 51) and were designed to enhance the ecological validity of the gait assessment by simulating real-world walking situations commonly encountered by older adults, rather than merely controlling speed through abstract commands (52). Each participant completed three acceptable trials at each speed, with a 2 min rest period between each condition.

### Data processing

**Figure 2:**
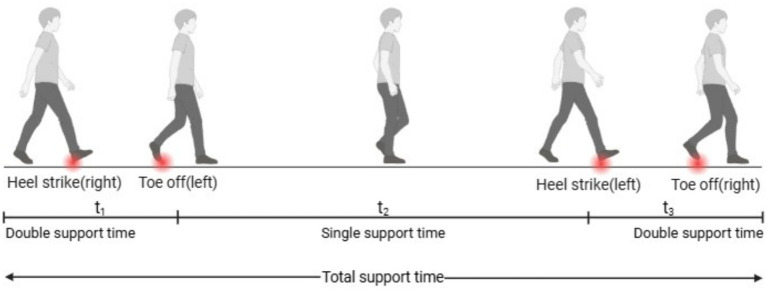
Diagram of gait events and temporal phases during walking. Red dots represented heel strike or toe-off events. *t*_1_ and *t*_3_ represented the double support time, *t*_2_ represented the single support time, and *t*_1_ + *t*_2_ + *t*_3_ represented the total support time.

**Figure 3:**
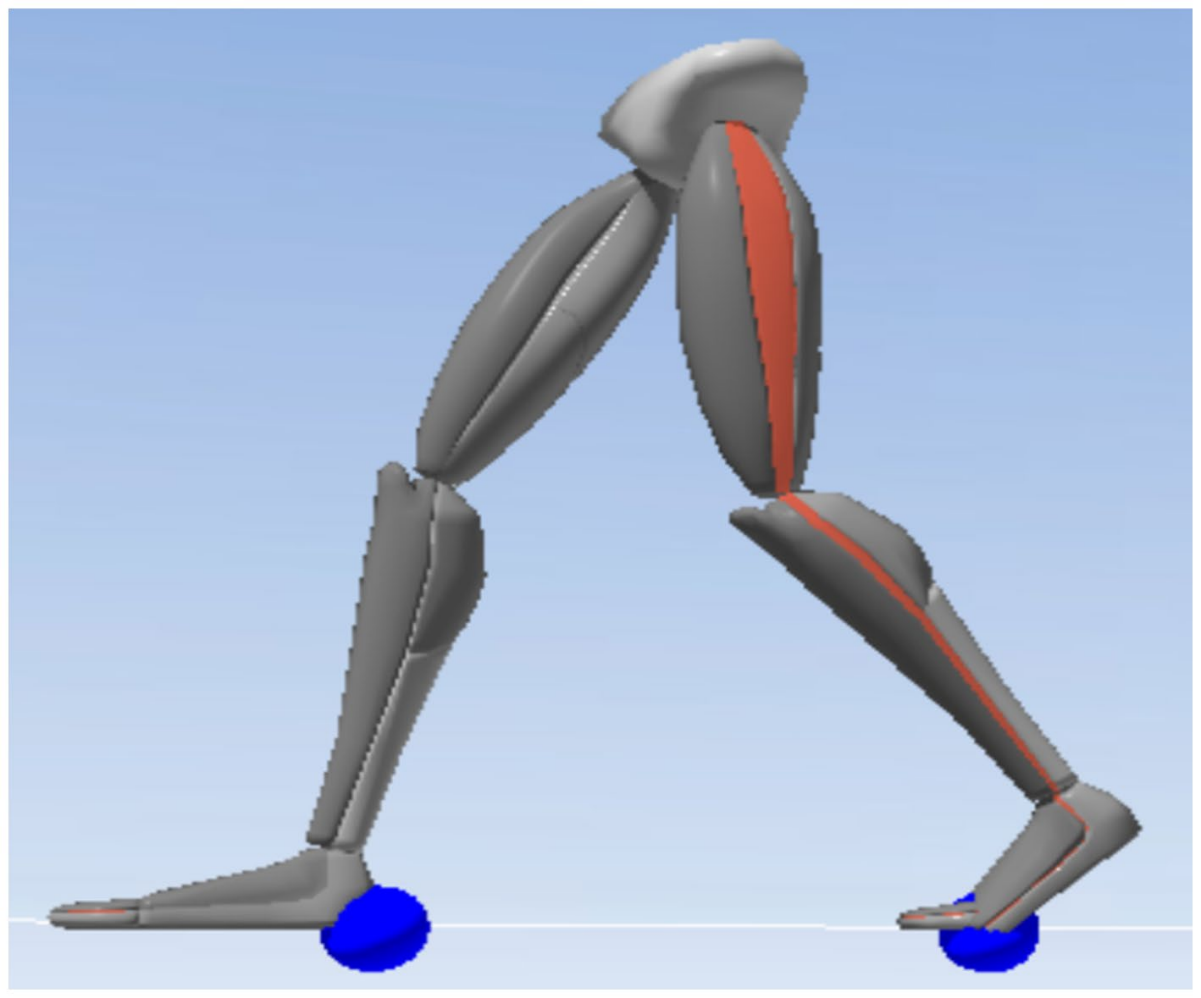
MVN analyze/animate interface (blue sphere indicates the contact point). A “contact point” referred to the virtual marker point in the analysis/animation interface of the Xsens MVN software, used to determine foot-ground contact events. Specifically, this blue sphere (contact point) was an algorithmically estimated instantaneous position of foot-ground contact based on inertial sensor data. It was employed to automatically identify gait events (such as heel strike and toe-off).

Data were processed using the Xsens MVN software. The stance phase was from heel strike to toe-off of the dominant side. The other two major gait events were toe-off and heel strike of the non-dominant side. These four primary gait events were used to define the total support time, single support time, and double support time (Figure 2). The main events of heel strike and toe-off were identified using the software’s contact function (53) (Figure 3).

The hip, knee, and ankle angles of both limbs were calculated as the Euler angle. Using the same method as in our prior study, interlimb (e.g., hip–knee) and intralimb (e.g., hip–hip) continuous relative phase (CRP) were calculated using joint angles and angular velocity of the sagittal plane (38).

First, angle (see Equation 1) and angular velocity (see Equation 2) were normalized using the equations below, scaling the angle values to a range of −1 to +1:

$${\theta_{i}}^{’}=\frac{2\times [\theta_{i}-\min(\theta_{i})]}{\max(\theta_{i})-\min(\theta_{i})}-1$$ (1)

$${\omega_{i}}^{’}=\frac{\omega_{i}}{\max|\omega_{i}|}$$ (2)

Here, i represented a data point in the stance phase; $\theta_{i}$ denoted the normalized angle at data point i ; $\theta_{i}$ indicated the raw angle at time point i ; ${\omega_{i}}^{’}$ referred to the normalized angular velocity at data point i ; $\omega_{i}$ represented the raw angular velocity at time point i .

Second, the phase angle for each joint at every time point during the cycle was determined by the following formula (see Equation 3):

$$\varphi (i)=\tan^{-1}[\frac{{\omega_{i}}^{’}}{{\theta_{i}}^{’}}]$$ (3)

Here, i was a data point in the stance phase, φ was the phase angle, $\omega_{i}$ was the normalized angular velocity, $\theta_{i}$ was the normalized angle.

Third, the CRP between two joints was calculated using the following formula (see Equation 4):

$$\mathrm{CRP}(i)=\varphi_{a}(i)-\varphi_{b}(i)$$ (4)

Here, $\varphi_{a}(i)$ was the phase angle of one joint at data point i . The phase angle of the other joint at the same point was $\varphi_{b}(i)$ .

Endpoint variability was calculated for the single support time, the double support time, and the total support time, while coordination variability was calculated for the intralimb CRP and interlimb CRP. For each walking trial, we excluded the initial gait cycles (acceleration phase) and the final gait cycles (deceleration phase). Finally, eight consecutive complete gait cycles were selected, and the standard deviation of each parameter was computed to quantify variability.

For intralimb CRP variability and interlimb CRP variability, the stance phase was further divided into two phases (54): (1) the braking phase (from initial contact to mid-stance) and (2) the propulsive phase (from mid-stance to toe-off). The CRP data were normalized to 100% of the stance phase. The analysis then proceeded in two steps: first, the standard deviation of the CRP across the eight trials for each percentage point was calculated, generating a time series of CRP variability. Second, the variability of stance and swing phases was computed from this derived time series as an average.

### Statistical analysis

The study used G*Power 3.1.9.7 software to calculate the minimum required sample size of 36 participants (55), with a medium effect size of 0.25 (56), a statistical power of 0.80 (57), and an *α* value of 0.05 (57).

Data were presented as mean ± standard deviation. Prior to conducting ANOVA, the normality of the data for each outcome variable across groups was examined using Shapiro–Wilk tests. The data met the assumption of normality, and given the robustness of ANOVA to minor deviations from normality with our sample size, parametric testing was deemed appropriate. The effects of frailty status and walking speed on support time variability, intralimb coordination variability, and interlimb coordination variability were determined using a two-way mixed ANOVA, with walking speed as a within-group factor and frailty status as a between-group factor. Sphericity was assessed using Mauchly’s test. Where the assumption of sphericity was violated, the Greenhouse–Geisser correction was applied. For significant interaction effects, post-hoc analyses were performed. All multiple comparisons were adjusted using the Bonferroni correction to identify specific significant differences. Effect sizes for significant effects are reported as partial eta squared (η_p_^2^), with values of 0.01, 0.06, and 0.14 interpreted as small, medium, and large effects, respectively. Data were incomplete for 13 of the 60 enrolled participants, primarily due to technical malfunctions of the IMU sensors (*n* = 9) or scheduling conflicts (*n* = 4). As these reasons were unrelated to participant characteristics, the missingness was considered Missing at Random (MAR), unlikely to introduce systematic bias. Statistical significance was set at 0.05. Statistical analyses were performed using SPSS version 26.0 (SPSS Inc., Chicago, IL, United States).

## Results

Table 1 summarized the demographic and clinical characteristics of the participants. Statistically significant differences were observed only for participant type (nursing homes vs. community, *p* = 0.004), while other variables showed no significant differences between groups (*p* > 0.05).

**Table 1:** Demographic and clinical characteristics of the participants.

Variables	Robust (*n* = 10)	Pre-frail (*n* = 20)	Frail (*n* = 17)	*P*-value
Age (years)	69.9 ± 7.3	75.3 ± 7.1	76.0 ± 9.5	0.053
Sex, male/female (*n*)	6/4	6/14	6/11	0.364
Height (cm)	164.8 ± 7.2	158.6 ± 9.2	160.6 ± 8.1	0.490
Weight (kg)	72.7 ± 8.6	64.8 ± 10.2	67.7 ± 14.1	0.348
BMI (kg/m^2^)	26.7 ± 2.1	25.7 ± 2.9	26.4 ± 4.9	0.648
Participant type, nursing homes/community (*n*)	0/10	12/8	10/7	0.004

The two-way mixed ANOVA revealed a significant interaction between group and speed on total support time variability (*F* = 2.894, *p* = 0.044, η_p_^2^ = 0.116). Post-hoc analysis revealed that the frail (*p* = 0.036) and pre-frail (*p* = 0.011) groups exhibited significantly greater total support time variability during fast walking compared to the robust group. In the frail group, total support time variability during fast walking was significantly higher than during slow walking (*p* = 0.015) (Table 2). In the pre-frail group, total support time variability during fast walking was significantly greater than during both preferred speed (*p* < 0.001) and slow walking (*p* < 0.001). In the robust group, no significant differences were found in total support time variability across the three walking speeds (all *p* > 0.05) (Table 2).

**Table 2:** Comparison of endpoint variability.

Variables (s)	Robust group (*n* = 10)			Pre-frail group (*n* = 20)			Frail group (*n* = 17)		
	Slow speed	Preferred speed	Fast speed	Slow speed	Preferred speed	Fast speed	Slow speed	Preferred speed	Fast speed
Total support time variability	0.021 ± 0.011	0.018 ± 0.009	0.017 ± 0.005^#*^	0.021 ± 0.011	0.019 ± 0.006	0.037 ± 0.026^a#*^	0.022 ± 0.017	0.024 ± 0.016	0.033 ± 0.014^a#*^
Single support time variability	0.022 ± 0.007	0.018 ± 0.009	0.020 ± 0.007	0.021 ± 0.009	0.019 ± 0.007	0.029 ± 0.020	0.032 ± 0.052	0.021 ± 0.017	0.025 ± 0.010
Double support time variability	0.018 ± 0.006	0.018 ± 0.007	0.020 ± 0.007	0.019 ± 0.007	0.018 ± 0.006	0.023 ± 0.016	0.031 ± 0.040	0.020 ± 0.021	0.026 ± 0.017

^a^ Significant difference compared to robust group; ^#^ Significant difference compared to slow speed; ^*^ Significantly difference compared to preferred speed.

The results of intralimb coordination variability showed that the knee-ankle variability during braking phase was significantly influenced by the interaction of group and speed (*F* = 3.098, *p* = 0.020, η_p_^2^ = 0.123). Post-hoc analysis revealed that during fast walking, the frail group (*p* = 0.014) exhibited significantly lower knee-ankle variability compared to the robust group. In the frail group, knee-ankle variability was significantly higher at preferred speed walking compared to slow walking (*p* = 0.016) and fast walking (*p* = 0.015). In the pre-frail group, knee-ankle variability was significantly greater at preferred speed walking compared to fast walking (*p* = 0.017). For the hip-knee coordination variability during propulsive phase, speed had a significant main effect (*F* = 4.099, *p* = 0.020, η_p_^2^ = 0.085). The variability was significantly higher during preferred speed (*p* = 0.011) and fast walking (p = 0.016) compared to slow walking (Table 3).

**Table 3:** Comparison of intralimb coordination variability.

Variables (°)		Robust group (*n* = 10)			Pre-frail group (*n* = 20)			Frail group (*n* = 17)		
		Slow speed	Preferred speed	Fast speed	Slow speed	Preferred speed	Fast speed	Slow speed	Preferred speed	Fast speed
Hip-knee coordination variability	Braking phase	29.30 ± 12.19	29.93 ± 10.98	30.12 ± 12.33	29.86 ± 15.66	30.13 ± 16.60	26.75 ± 14.77	19.72 ± 14.67^b^	25.65 ± 16.82	20.96 ± 14.92
	Propulsive phase	15.45 ± 4.02^*^	18.79 ± 7.67	17.42 ± 5.93^#^	14.78 ± 4.70^*^	15.99 ± 5.05	16.39 ± 5.08^#^	12.86 ± 3.16^*a^	14.23 ± 4.09^a^	14.53 ± 4.78^#^
Knee-ankle coordination variability	Braking phase	28.88 ± 12.55	27.92 ± 12.86	37.64 ± 21.28	29.97 ± 19.72	35.62 ± 20.52	25.61 ± 16.29^*^	21.23 ± 13.40	31.38 ± 22.04^#^	20.29 ± 14.77^a*^
	Propulsive phase	21.17 ± 7.01	23.77 ± 5.43	22.62 ± 6.24	22.08 ± 5.01	23.96 ± 4.92	21.49 ± 5.40	18.87 ± 6.20	20.33 ± 3.49	21.68 ± 5.20

^a^ Significant difference compared to robust group; ^#^ Significant difference compared to slow speed; ^*^ Significantly difference compared to preferred speed.

The results of interlimb coordination variability showed that the hip-hip variability during propulsive phase was significantly influenced by the interaction between group and speed (*F* = 4.495, *p* = 0.002, η_p_^2^ = 0.170). Post-hoc analysis showed that both the frail (*p* < 0.001) and pre-frail (*p* < 0.001) groups exhibited significantly lower variability than the robust group during fast and slow walking. In the robust group, slow walking resulted in significantly lower variability compared to preferred (*p* < 0.001) and fast walking (*p* < 0.001). Knee–knee variability during propulsive phase (*F* = 112.678, *p* < 0.001, η_p_^2^ = 0.837) and ankle-ankle variability during braking phase (*F* = 4.669, *p* = 0.014, η_p_^2^ = 0.175) were significantly influenced by the group, with the frail and pre-frail groups showing lower variability than the robust group (*p* < 0.01). Speed also significantly affected knee–knee variability during propulsive phase (*F* = 4.253, *p* = 0.017, η_p_^2^ = 0.088) and ankle-ankle variability during braking phase (*F* = 5.457, *p* = 0.006, η_p_^2^ = 0.110). Knee–knee variability was significantly lower during slow walking than fast walking (*p* = 0.006). Ankle-ankle variability was significantly higher during preferred speed than fast (*p* = 0.011) and slow walking (*p* = 0.008). Additionally, hip-hip variability during braking phase (*F* = 306.495, *p* < 0.001, η_p_^2^ = 0.933) and ankle-ankle variability during propulsive phase (*F* = 23.017, *p* < 0.001, η_p_^2^ = 0.511) were significantly influenced by group, both the frail and pre-frail groups exhibited significantly lower variability than the robust group (all *p* < 0.001) (Table 4).

**Table 4:** Comparison of interlimb coordination variability.

Variables (°)		Robust group (*n* = 10)			Pre-frail group (*n* = 20)			Frail group (*n* = 17)		
		Slow speed	Preferred speed	Fast speed	Slow speed	Preferred speed	Fast speed	Slow speed	Preferred speed	Fast speed
Hip-Hip coordination variability	Braking phase	36.72 ± 17.55	39.47 ± 8.44	43.34 ± 8.02	6.73 ± 1.71^a^	5.86 ± 1.74^a^	6.42 ± 1.62^a^	7.60 ± 2.56^a^	8.69 ± 5.98^a^	9.42 ± 6.40^a^
	Propulsive phase	42.35 ± 5.21^*^	49.60 ± 2.98^#^	49.70 ± 1.22^#^	14.37 ± 4.14^a*^	13.79 ± 3.13^a^	15.47 ± 4.08^a#^	14.49 ± 4.76^a*^	14.95 ± 4.72^a^	15.63 ± 4.90^a#^
Knee–knee coordination variability	Braking phase	48.41 ± 13.20	47.95 ± 11.58	50.22 ± 15.28	29.97 ± 19.72	43.68 ± 18.24	44.89 ± 13.95	38.55 ± 19.87	40.30 ± 17.68	39.68 ± 16.03
	Propulsive phase	40.37 ± 6.29	41.51 ± 7.18	46.44 ± 5.80^#^	17.54 ± 4.24^a^	18.63 ± 5.89^a^	19.51 ± 7.43^#a^	16.55 ± 5.36^a^	19.18 ± 5.43^a^	17.36 ± 6.32^#a^
Ankle-ankle coordination variability	Braking phase	38.92 ± 15.36	43.49 ± 9.87^#^	44.63 ± 12.63^*^	31.46 ± 15.57^a^	38.91 ± 11.01^a#^	26.63 ± 13.70^a*^	28.09 ± 12.30^a^	38.18 ± 14.33^a#^	29.40 ± 12.46^a*^
	Propulsive phase	44.86 ± 3.38	46.21 ± 4.06	45.31 ± 4.23	32.85 ± 6.33^a^	29.89 ± 7.78^a^	35.04 ± 8.45^a^	33.75 ± 8.69^a^	32.21 ± 8.37^a^	36.42 ± 7.72^a^

^a^ Significant difference compared to robust group. ^#^ Significant difference compared to slow speed. ^*^ Significantly difference compared to preferred speed.

## Discussion

This study investigated how frailty influences endpoint variability and coordination variability in older adults during walking at different speeds. The findings supported our two hypotheses. Firstly, the first hypothesis was that frailty differentially affects endpoint and coordination variability. Specifically, compared to the robust group, frail and pre-frail individuals showed greater total support time variability and lower coordination variability across several joint pairs: specifically, during the braking phase in hip-hip, ankle-ankle, and knee-ankle, and during the propulsive phase in hip-hip, knee–knee, and ankle-ankle.

The findings supported Hamill et al.’s perspective that endpoint variability and coordination variability may play different roles in human movement (25). Coordination variability reflects motor flexibility and adaptability; higher variability means a wider range of coordination strategies in response to changing environments and tasks (25). In contrast, endpoint variability was commonly used to assess movement precision (58), with greater variability typically suggesting less precise or stable motor control.

Decreased coordination variability in frail older adults may suggest reduced neuromuscular control, which leads to restricted freedom of movement and movement rigidity. These changes weaken the ability of individuals to adapt to perturbations of gait adjustments and ultimately lead to the decline of movement accuracy and stability (25). This result was consistent with our previous findings that athletes with higher skill levels exhibited greater coordination variability and lower endpoint variability during running compared to non-athletes, highlighting how long-term training enhances adaptive movement patterns and stable movement outcomes (38).

The reduced coordination variability in frail adults may also partly explain the biomechanical mechanism of their higher fall risk (59). That is, frailty leads to a decrease in coordination variability during walking, resulting in insufficient adaptability to movement tasks and the environment, ultimately leading to falls. Similarly, one study revealed that the faller group exhibited significantly lower coordination variability during the stance phase compared to the non-faller group (32), further supporting the link between coordination variability deficits and fall risk.

These findings highlighted the potential of gait variability analysis for early detection of frailty and development of targeted intervention strategies in older adults. Specifically, total support time variability and coordination variability effectively distinguished frail from robust older adults. Notably, frailty’s effects varied by gait phase and speed: while its effects on total support time variability and ankle-knee variability during braking phase were only observed during fast walking, hip-hip variability during propulsive phase was influenced across both fast and slow speeds.

For interlimb and intralimb coordination variability, frailty primarily influenced interlimb coordination variability, with only minimal effects on intralimb knee-ankle coordination variability during the braking phase. This suggested that declining neuromuscular control in older adults may first disrupt interlimb coordination patterns. Our findings were relatively consistent with those of a previous study: during walking in healthy young adults, inter-limb coordination patterns in the lower extremities were more sensitive to changes in treadmill speed than intra-limb coordination, demonstrating more significant adjustments in coordination patterns (60). In contrast, intra-limb coordination variability remained relatively stable within the gait pattern (60). Future research should explore the distinct roles of intralimb and interlimb coordination variability in gait control across populations, particularly their sensitivity and temporal sequence in neuromuscular decline or developmental disorders.

Both coordination variability and endpoint variability showed similar patterns, with significant differences mainly observed between the robust group and the pre-frail/frail groups, but not between the pre-frail and frail groups. However, a previous study revealed that during fast walking, frail older adults exhibited higher stride time variability compared to pre-frail older adults, suggesting that stride time variability is a key gait parameter for distinguishing between pre-frail and frail states (20). The discrepancy between the findings of the two studies may be attributed to the differences in the variability measures used. To gain a more comprehensive understanding of gait characteristics across different frailty states, it is recommended that future research incorporate variability measures from multiple dimensions for integrated analysis. Furthermore, other studies found that frail older adults exhibited significantly slower gait speed, longer double support time and total support time compared with the pre-frail older adults (61), and lower peak hip adduction angles, hip and knee flexion angles, and knee and ankle internal rotation angles compared to pre-frail and robust older adults (62). These discrepancies suggested that while mean gait values may differentiate pre-frailty from frailty, variability metrics might better distinguish robust individuals from those in pre-frail/frail states. The lack of variability differences between pre-frail and frail groups could also relate to the assessment tool used. The KCL scale evaluates multidimensional factors (e.g., cognitive, psychological, and social function), implying that non-physical factors may play a more critical role in distinguishing pre-frailty from frailty.

Secondly, this study supported our second hypothesis that walking speed influenced gait variability. Specifically, fast walking increases endpoint variability, while coordination variability varies depending on the type of measure used. Notably, frail and pre-frail older adults exhibited greater total support time variability during fast walking, aligning with prior findings that fast walking increases endpoint variability (e.g., single support time, double support time, and stance time) compared to slow and preferred speed walking (39). Although we did not objectively measure walking speed in this study, it is well established that gait speed progressively declines across the frailty spectrum, with significant differences typically observed between pre-frail and frail groups (63–66). As such, the similar variability outcomes observed between these groups in our study are unlikely to be explained by comparable walking speeds. Since fast walking imposes higher biomechanical demands (67, 68), diminished physical function in frail individuals may compromise movement stability, leading to elevated variability. Furthermore, knee-ankle and ankle-ankle variability significantly increase during braking phase of preferred speed walking, whereas hip-knee and hip-hip variability significantly increase during propulsive phase of both preferred speed and fast walking. The coordination patterns between joints and their variability provided insights into neuromuscular control during functional movements (30, 69). This suggested that during preferred speed walking, coordination patterns between distal and proximal joints in older adults may retain sufficient flexibility to adapt to environmental and task demands. However, fast walking, as a more challenging task, increases the difficulty of motor control and places higher demands on the neuromuscular-skeletal system. Therefore, greater coordination variability may be required to provide enhanced flexibility under these conditions (36). Notably, during fast walking, only hip-knee and hip-hip variability were significantly greater in propulsion phase, with no changes in knee-ankle or ankle-ankle variability during braking phase. This suggested that while faster walking requires greater flexibility (70), age-related declines in neuromuscular control may limit older adults’ ability to adapt by adjusting distal joint coordination (71, 72). Consequently, this impairment could contribute to adverse outcomes during fast walking. These findings highlight gait speed modulation as a potential rehabilitation target for frail older adults, for instance, through personalized speed training to improve joint coordination control. Further research should validate the effects of walking speed on variability to optimize intervention strategies.

This study has several limitations. First, the cross-sectional design limits causal interpretations. Future longitudinal studies with larger samples are needed to better understand the variability-frailty relationship in older adults. Second, the inclusion of participants from both community and nursing home settings may introduce heterogeneity due to inherent differences in living environment, support systems, and daily routines. Third, the use of the KCL tool to assess frailty in this study may limit the comparability of our findings with other studies that used different tools, such as the FFI or CFS. Fourth, the endpoint variability analysis in this study only focused on support time variability (total, single, and double), without covering fluctuations in other spatiotemporal parameters (such as step length, step width, and gait speed). Future studies will incorporate optical motion capture technology to enable more reliable spatial gait analysis and more comprehensively assess the impact of frailty on various aspects of gait control. Additionally, further research should investigate diverse movement patterns and populations to clarify the distinct contributions of endpoint and coordinative variability to locomotor performance.

## Conclusion

Frailty differentially affected endpoint variability and coordination variability in older adults’ gait. Frail older adults exhibited lower coordination variability compared to their robust older adults, particularly in interlimb coordination variability, indicating reduced adaptability and flexibility to task and environmental demands, which may lead to less stable walking control. Compared to fast or slow walking, during preferred speed walking, frail and pre-frail older adults exhibited greater coordination variability in distal joint during braking phase, demonstrating a more flexible coordination pattern. The limited changes in distal joint coordination during braking phase may contribute to adverse outcomes during fast walking. This study used a cross-sectional design with a limited sample; future longitudinal studies are needed for verification, and more gait parameters and population differences should be explored.

## Data Availability

The original contributions presented in the study are included in the article/supplementary material, further inquiries can be directed to the corresponding author.
